# Trends in Geographic Disparities in Access to Ambulatory Surgery Centers in New York, 2010 to 2018

**DOI:** 10.1001/jamahealthforum.2022.3608

**Published:** 2022-10-14

**Authors:** Abhinaba Chatterjee, Troy B. Amen, Sariah Khormaee

**Affiliations:** 1 Weill Cornell Medical College, New York, New York; 2 Hospital for Special Surgery, New York, New York

## Abstract

This cross-sectional analysis evaluates trends in the density, volume, and utilization of ambulatory surgery centers by neighborhood socioeconomic status.

## Introduction

Nearly half of all outpatient procedures occur in ambulatory surgery centers (ASCs).^1^ Prior studies have reported racial and socioeconomic disparities in the utilization of ASCs, raising concern that structural barriers may be limiting access to outpatient surgery among patients who are less advantaged.^2^ However, the data regarding disparities in the geographic distribution of ASCs are limited, and how these disparities have changed over time is, to our knowledge, unknown. The aim of this study was to evaluate trends in the density, volume, and utilization of ASCs by neighborhood socioeconomic status.

## Methods

This study was approved by the institutional review board at Weill Cornell Medicine. The study followed the Strengthening the Reporting of Observational Studies in Epidemiology (STROBE) reporting guidelines for cross-sectional studies.

We conducted a retrospective cross-sectional analysis using data from the New York Statewide Planning and Research Cooperative System (SPARCS), which prospectively collects data on every ambulatory surgery visit in the state. We focused the analysis on patients who underwent ambulatory arthroscopy, cataract excision, cystoscopy, cholecystectomy, or endoscopy in 2010 to 2018, using *Current Procedural Terminology* codes that were previously described (eMethods 1 in the Supplement).^2,3,4^ Facilities were designated as ASCs or hospital outpatient departments (HOPDs) using data obtained from the SPARCS website.

A previously validated socioeconomic advantage score (SES)^5^ was calculated at the county and zip code−level using data from the American Community Survey (2010-2018). This summary score comprised 6 variables that measured wealth, education, and occupation (eMethods 2 in the Supplement). Regions were stratified into 3 ascending tertiles, each of which contained 33% of the state population, ranked by SES.

Changes in ASC density, volume, and utilization over time were analyzed by fitting a linear term for calendar year in adjusted Poisson regression models. An interaction term between an SES tertile and year was included to test differences in trends over time (more details in eMethods 3 in the Supplement). The coefficients from these models were used to estimate annual percentage changes (APC) for each outcome. A 2-sided *P* value < .05 determined statistical significance. Data analyses were conducted from March 17 to August 16, 2022, using Stata-MP, version 17.0 (StataCorp, LLC).

## Results

From 2010 to 2018, ASC density, volume, and utilization increased across all county tertiles. Trends in ASC location and per-capita volume are summarized in the Figure.

**Figure.**
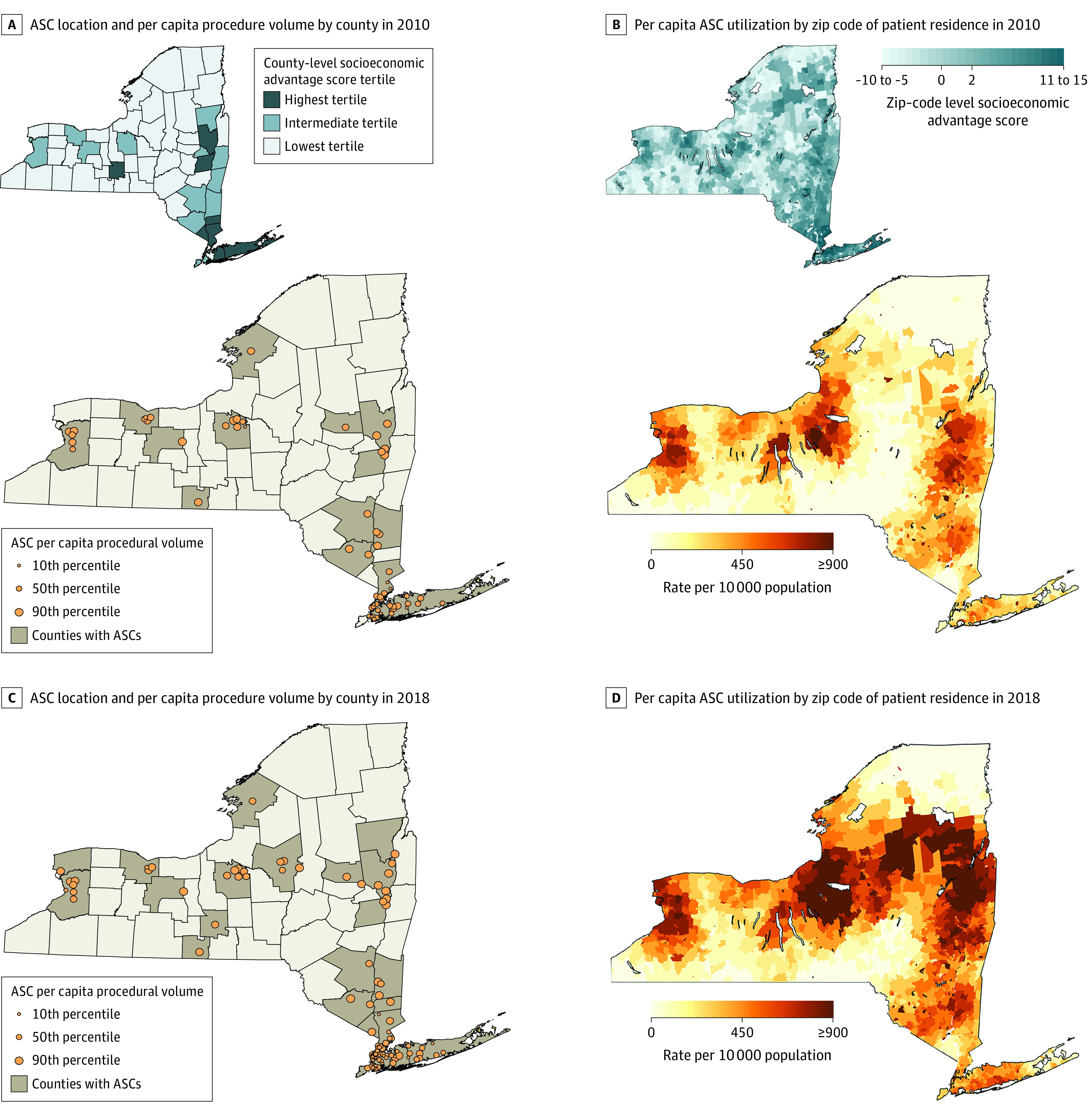
Volume and Utilization of Ambulatory Surgery Centers (ASCs) in New York State, 2010 to 2018 A and C, Location and volume of procedures performed in ASCs, by county population in 2010 and 2018, demonstrating the geographic distribution of ASC locations, weighted by the procedural volume of each ASC. B and D, Number of patients residing in each zip code who received a procedure in an ASC, by population of that zip code, 2010 and 2018, respectively. Socioeconomic advantage score tertile is also shown at the county level; raw socioeconomic advantage scores are shown at the zip code−level to provide a more detailed depiction of geographic distribution.

There were persistent differences in the geographic distribution of ASCs by SES tertile, with more affluent counties having a higher number of ASCs per capita throughout the study period (Table). There were increasing disparities between the least and most advantaged counties in ASC volume (APC, 9.4% vs 10.8%; *P* < .001 for interaction) and utilization (APC, 10.8% vs 13.0%; *P* < .001 for interaction). The number of HOPDs did not change over time; however, there were declines in HOPD volume (APC, −2.8% vs 0.1%) and utilization (APC, −2.0% vs 0.5%) that were most significantly associated with less advantaged counties (*P* < .001 for interaction).

**Table.** County-Level Summary Statistics for Ambulatory Surgery Centers by Socioeconomic Status Tertile, 2010 to 2018^a^

Characteristic	Lowest tertile					Intermediate tertile						Highest tertile				
	2010-2012	2013-2015	2016-2018	RD (95% CI)	APC^b^ (95% CI)	2010-2012	2013-2015	2016-2018	RD (95% CI)	APC (95% CI)		2010-2012	2013-2015	2016-2018	RD (95% CI)	APC (95% CI)
**Demographic characteristics of ASC patients**																
Age, mean (SD), y	59.8 (0.03)	59.6 (0.02)	59.7 (0.02)	NA	NA	60.1 (0.02)	60.2 (0.02)	59.8 (0.02)	NA		NA	58.5 (0.03)	59.5 (0.02)	59.3 (0.02)	NA	NA
Race and ethnicity^c^																
Asian or Pacific Islander	8548 (3.8)	16 664 (4.5)	47 782 (10.3)	NA	NA	6391 (1.6)	12 687 (2.6)	21 920 (3.6)	NA		NA	5959 (2.3)	14 626 (3.3)	21 244 (3.7)	NA	NA
Hispanic	21 952 (9.7)	81 975 (22.1)	90 591 (19.4)	NA	NA	8236 (2.0)	24 575 (4.9)	30 621 (4.9)	NA		NA	32 616 (12.2)	42 230 (9.4)	66 126 (11.4)	NA	NA
Non-Hispanic Black	25 276 (11.1)	39 561 (10.7)	44 302 (9.5)	NA	NA	22 031 (5.4)	49 381 (9.8)	63 942 (10.3)	NA		NA	14 351 (5.4)	29 046 (6.5)	40 146 (6.9)	NA	NA
Non-Hispanic White	99 715 (43.7)	190 091 (51.1)	224 712 (48.2)	NA	NA	223 700 (54.0)	331 164 (65.4)	400 631 (64.1)	NA		NA	150 580 (56.1)	261 898 (57.9)	340 705 (58.6)	NA	NA
Other^d^	1663 (0.8)	5676 (1.6)	4075 (0.9)	NA	NA	6348 (1.6)	3139 (0.7)	5208 (0.9)	NA		NA	855 (0.4)	4528 (1.0)	5212 (0.9)	NA	NA
Source of payment																
Private	147 444 (66.3)	232 090 (62.3)	288 520 (61.3)	NA	NA	286 897 (69.2)	368 945 (72.8)	443 221 (70.7)	NA		NA	196 513 (73.2)	336 568 (74.3)	423 270 (72.6)	NA	NA
Medicare	52 807 (23.7)	96 893 (26)	116 860 (24.8)	NA	NA	104 504 (25.2)	130 740 (25.8)	166 931 (26.6)	NA		NA	71 339 (26.6)	131 117 (29.0)	168 603 (28.9)	NA	NA
Medicaid	18 010 (8.1)	47 563 (12.8)	91 660 (19.5)	NA	NA	10 945 (2.6)	36 374 (7.2)	66 167 (10.6)	NA		NA	6351 (2.4)	22 362 (4.9)	51 894 (8.9)	NA	NA
**Population-based ASC trends**																
No. of ASCs/million person-years	2.3	2.9	3.5	1.2 (1.0 to 1.5)	9.5 (–0.2 to 20.0)	6.1	6.8	8.0	1.7 (1.4 to 1.8)		7.7 (–0.2 to 16)	9.7	12.6	13.9	3.8 (3.5 to 4)	7.4 (2.3 to 12.8)
ASC volume/10 000 person-years^e^	7.3	13.7	16.0	7.8 (7.7 to 7.8)	9.4 (8.6 to 10.2)	25.4	29.2	33.7	10.8 (10.8 to 10.9)		4.9 (4.4 to 5.4)	22.9	32.4	40.1	21.5 (21.5 to 21.6)	10.8 (10.1 to 11.5)^f^
ASC utilization/10 000 person-years^g^	11.9	19.9	25.1	14.2 (14.2 to 14.3)	10.8 (10.4 to 11.2)	21.0	25.7	31.7	13.6 (13.5 to 13.6)		8.5 (8.0 to 9.0)	13.8	23.2	29.9	21.4 (21.3 to 21.5)	13.0 (12.1 to 13.9)^f^
**Population-based HOPD trends**																
No. of HOPDs/million person-years	15.5	16.1	15.3	–0.8 (–1.0 to –0.5)	–0.9 (–3.8 to 2.1)	10.0	10.8	10.2	0.5 (0.3 to 0.6)		0.1 (–3.9 to 4.4)	11.8	12.9	12.3	–0.9 (–1.1 to –0.8)	–2.1 (–5.3 to 1.3)
HOPD volume/1000 person-years	39.5	37.0	34.9	–7.7 (–7.8 to –7.6)	–2.8 (–2.9 to –2.7)	39.5	38.0	35.8	–4.3 (–4.3 to –4.2)		–2.3 (–2.5 to –2.0)	47.9	48.2	47.7	–1.4 (–1.5 to –1.4)	0.1 (–0.3 to 0.6)^f^
HOPD utilization/1000 person-years	48.7	46.0	44.8	–7.2 (–7.2 to –7.1)	–2.0 (–2.1 to –1.9)	37.1	36.8	35.2	–3.1 (–3.1 to –3.0)		–0.4 (–0.7 to –0.1)^h^	33.9	34.2	34.0	–0.1 (–0.1 to 0)	0.5 (0.1 to 1.0)^f^
**Utilization by clinical specialty**																
Upper GI endo-/colonoscopy																
ASC utilization/10 000 person-years	56.8	110.5	134.3	89.1 (88.7 to 89.4)	12.9 (6.9 to 19.2)	106.1	130.6	170.4	81.5 (81.2 to 81.7)		8.9 (4.3 to 13.8)	80.6	134.8	181.7	132 (131.6 to 132.5)	14.5 (8.1 to 21.3)^f^
HOPD utilization/10 000 person-years	245.8	227.8	224.0	–37.6 (–37.7 to –37.6)	–1.9 (–3.3 to –0.5)	182.0	177.4	164.3	–25.8 (–26 to –25.6)		–0.5 (–4 to 3.3)	134.5	134.4	139.1	5.8 (5.8 to 5.9)	0.1 (–3.4 to 3.9)^f^
Cholecystectomy procedure																
ASC utilization/10 000 person-years	0.2	0.3	0.2	–0.1 (–0.1 to 0)	–8.1 (–13.6 to –2.3)	1.1	0.9	0.7	–0.4 (–0.5 to –0.4)		–8.4 (–15.5 to –0.6)	0.5	0.5	0.4	–0.1 (–0.1 to 0)	–9.9 (–18 to –1)
HOPD utilization/10 000 person-years	24.2	25.8	24.7	0.2 (0.2 to 0.3)	–0.8 (–1.9 to 0.5)	23.6	24.3	23.5	1.1 (1.1 to 1.2)		–0.2 (–2.0 to 1.8)	18.9	20.1	20.6	2.5 (2.5 to 2.6)	0.6 (–2.0 to 3.4)
Cataract excision/lens insertion																
ASC utilization/10 000 person-years	45.4	67.3	84.4	43.5 (43.3 to 43.7)	13.1 (8.6 to 17.6)	71.2	89.8	103.9	38.9 (38.8 to 39)		10.3 (5.7 to 15.1)	35.2	72.3	90.3	74.2 (73.8 to 74.6)	15.5 (8.9 to 22.1)^f^
HOPD utilization/10 000 person-years	72.0	68.2	65.0	–15.8 (–16.0 to –15.7)	–2.8 (–4.9 to –0.7)	43.4	42.5	40.2	–6.4 (–6.5 to –6.3)		–2.4 (–6.7 to 2.3)	57.3	59.8	57.6	–0.1 (–0.2 to –0.1)	1.5 (–2.0 to 5.2)^f^
Arthroscopy procedure																
ASC utilization/10 000 person-years	15.3	17.5	20.8	7.3 (7.3 to 7.4)	5.8 (2.3 to 9.3)	22.7	25.1	29.4	10.1 (10 to 10.2)		3.4 (0.1 to 6.9)	18.9	21.2	21.7	5.6 (5.6 to 5.7)	2.3 (–4.3 to 9.4)
HOPD utilization/10 000 person-years	38.7	38.4	33.7	–7.5 (–7.6 to –7.4)	–1.4 (–3 to 0.3)	31.0	29.8	26.3	–7.0 (–7.1 to –6.9)		–1 (–4.3 to 2.6)	42.1	39.5	34.7	–10.5 (–10.6 to –10.4)	–0.7 (–3.8 to 2.5)
Cystoscopy procedure																
ASC utilization/10 000 person-years	1.9	3.0	4.2	2.3 (2.2 to 2.4)	7.7 (1.8 to 14)	8.9	10.4	13.2	5.5 (5.5 to 5.6)		4.7 (–5.5 to 15)	2.4	3.5	4.9	2.8 (2.7 to 2.8)	6.8 (–2.8 to 17.4)
HOPD utilization/10 000 person-years	107.4	101.8	101.8	–11.9 (–12.0 to –11.8)	–2.4 (–4.1 to –0.8)	92.7	95.9	98.5	6.4 (6.4.0 to 6.5)		0.8 (–0.9 to 2.7)	88.5	90.3	88.9	0.3 (0.2 to 0.3)	0.7 (–1 to 2.6)

Abbreviations: APC, annual percent change; ASC, ambulatory surgery center; GI, gastrointestinal; HOPD, hospital outpatient department; NA, not applicable; RD, rate difference; SES, socioeconomic advantage score.

^a^ All values are represented as No. (%), unless otherwise specified.

^b^ Derived from coefficients of Poisson regression models adjusted for age, sex, race and ethnicity, and comorbidity burden.

^c^ Race and ethnicity were recorded by the facility treating each patient, and then reported to the New York Statewide Planning and Research Cooperative System.

^d^ Includes Native American and multiracial patients.

^e^ Per-capita ASC procedural volume refers to the number of procedures performed within an ASC physically located in a specified county divided by the population of that county.

^f^ Denotes interaction between calendar year and high vs low SES tertile.

^g^ Per-capita ASC utilization refers to the number of patients residing in a county that received a procedure in an ASC divided by the population of that county.

^h^ Denotes interaction between calendar year and intermediate vs low SES tertile.

Trends varied for each procedure type. Utilization of arthroscopy, cystoscopy, and cholecystectomy were consistent across SES tertile, whereas increases in ASC volume and utilization for cataract excision and upper GI endoscopy or colonoscopy differed by SES tertile (*P* < .001 for interaction; Table).

## Discussion

We found a persistent geographic disparity in the counties in which ASCs were emerging, as well as widening disparities in the volume and utilization of ASCs. There were also substantial declines in HOPD volume and utilization associated with less advantaged counties. Limitations of the study analysis included the focus on New York state, therefore, the findings may not generalize to other states.

Expansion of ASCs may be limited in counties with low SES because of several issues that facility owners may be hesitant to accept: lower value reimbursements from public insurance payers (more common in less affluent neighborhoods), competition from hospitals as the established health care source in these settings, and regulations—eg, certificates of need—that may delay efforts to open new facilities.^6^ The decline in HOPD utilization occurred in counties with low SES despite lower increases in ASC volume, and may indicate that hospitals in these regions are more vulnerable to competition from ASCs. These study findings suggest that patients from resource poor settings may face growing geographic barriers when pursuing ambulatory care. Further research is necessary to better understand the factors that make less advantaged communities more susceptible to these trends.

## Notes

Supplement. eMethods 1. CPT Codes for Ambulatory Procedures eMethods 2. Socioeconomic Advantage Score eMethods 3. Modeling
