# Supplementary material for: Trends in Geographic Disparities in Access to Ambulatory Surgery Centers in New York, 2010 to 2018
Source: JAMA Health Forum. 2022 Oct 14;3(10):e223608. doi: 10.1001/jamahealthforum.2022.3608 (PMC9568805; doi:10.1001/jamahealthforum.2022.3608)
Supplement: Supplement. — eMethods 1. CPT Codes for Ambulatory Procedures eMethods 2. Socioeconomic Advantage Score eMethods 3. Modeling [file jamahealthforum-e223608-s001.pdf]

Supplemental Online Content

Chatterjee A, Amen TB, Khormaee S. Trends in geographic disparities in access to ambulatory surgery centers in New York, 2010 to 2018. *JAMA Health Forum.* 2022;3(10):e223608. doi:10.1001/jamahealthforum.2022.3608

eMethods 1. CPT Codes for Ambulatory Procedures

eMethods 2. Socioeconomic Advantage Score

eMethods 3. Modeling

This supplemental material has been provided by the authors to give readers additional information about their work.

© 2022 Chatterjee A et al. *JAMA Health Forum.*

eMethods 1. CPT Codes for Ambulatory Procedures

To identify procedures of specific clinical specialties, the following CPT codes listed below were used. These procedures are consistently reported as being the most common ambulatory procedures in the United States[1] and have been evaluated in prior studies.[1-4] In the present study they accounted for 62.4% of all ambulatory volume, thus allowing us to capture trends in overall ambulatory procedure use while eliminating noise from other less common procedures. These procedures are also diverse with respect to the nature of their indications and clinical subspecialties that perform them.

**Clinical Subspecialty Specific CPT Codes**	
Gastroenterology:	Upper GI endoscopy (43235, 43239, & 43248) and colonoscopy (45378, 45380, 45383-45384)
General surgery:	Cholecystectomy (47562)
Ophthalmology:	Cataract removal/lens insertion (66982, 66984, 66821)
Orthopedics:	Shoulder arthroscopy (29805-29828), knee arthroscopy (29877-29889), and hip arthroscopy (29860-29863)
Urology:	Cystoscopy (52xxx) and (55700)

© 2022 Chatterjee A et al. *JAMA Health Forum.*

eMethods 2. Socioeconomic Advantage Score

We calculated a previously validated socioeconomic advantage score (SES) for every county and zip-code in NY state.[5] Data for each region were obtained from American Community Survey tables that are made publicly available by the US Census. The summary score was comprised of six variables: 1) the log median household income; 2) log median value of housing units; 3) percentage of households receiving interest, dividend, or rental income; 4) the percentage of adults who had completed high school; 5) the percentage who had completed college; and 6) the percentage of individuals employed in managerial or professional specialty occupations. For each zip code or county, a summary score was calculated by adding the z-scores for each of these 6 variables. These scores are calculated relative to the rest of the United States; therefore, an SES of 0 represents the average in the country.

County-level Summary Statistics by Socioeconomic Status Tertile.[1]

	**Lowest tertile**	**Intermediate tertile**	**Highest tertile**
Number of counties	40	13	9
County socioeconomic status characteristics			
Wealth/Income			
Median household income ($)	54,742 (51,096 - 54,387)	64,738 (59,787 - 69,689)	91,108 (80,839 - 99,825)
Median value of housing units ($)	119,650 (101,700 - 139,900)	193,800 (160,000 - 263,700)	386,800 (251,200 - 474,800)
Households with interest, dividends or net rental income, (%)	20.5 (19.3 - 21.7)	23.2 (20.9 - 25.4)	27.8 (25.8 - 29.7)
Education			
Adult residents who completed ≥ high school, (%)	88.4 (87.3 - 90.1)	90.5 (90.3 - 91.4)	91.3 (87.9 - 93.0)
Adult residents who completed ≥ college, (%)	22.3 (19.6 - 24.1)	33.1 (31.7 - 34.6)	41.4 (39.9 - 50.2)
Occupation			
Employed residents with management, professional, and related occupations, (%)	32.8 (31.9 - 33.7)	39.7 (38.4 - 40.9)	47.1 (43.0 - 51.1)
Socioeconomic status advantage score (SES)	1.06 (-0.13 – 1.71)	5.20 (4.62 – 5.70)	9.95 (8.94 – 11.11)

Abbreviations: IQR, interquartile range
[1] All values are represented as median, (IQR)

© 2022 Chatterjee A et al. *JAMA Health Forum.*

eMethods 3. Modeling

Definition of Outcomes

1) ASC Density: Number of ASCs per capita operating in a given region
2) ASC Procedural Volume: No. of procedures performed in ASCs within a given region per capita
3) ASC Utilization: No. of patients residing in a region that received a procedure in an ASC per capita

Poisson Regression Models

Three separate Poisson regression models were used to calculate rate ratios estimating the association between each outcome and SES tertile. These models predicted 1) the number of ASCs operating in a region per capita, 2) the number of procedures performed in ASCs within a region (irrespective of patient residence) per capita, and 3) the number of patients residing within a region that received a procedure within an ASC (irrespective of ASC location) per capita. The association between time and each outcome was evaluated by including calendar year as a covariate in the Poisson models. The time variable was operationalized in a continuous fashion. We felt this selection was justified since the data followed a Poisson distribution and the model chosen had a lower score on the Akaike information criterion relative to other models such as linear and negative binomial regression, indicating a better fit. There were no signs of deviance, non-normality, or heteroscedasticity noted during diagnostic evaluations of the residuals.

Each model was adjusted for multiple region- and time-specific covariates including age, sex, race/ethnicity, and comorbidity burden. Region- and time-specific comorbidity burdens were derived from estimates using all patients in the cohort (including the HOPD group).

The hypothesis that disparities in access to ASCs were widening was formally evaluated using two approaches. First, the absolute difference in annual percentage changes for each outcome was assessed to measure the difference in relative growth between each SES tertile. Finally, we calculated a formal test statistic by including interaction terms between SES tertile and calendar year in our Poisson models. Below is a formal model statement outlining our approach:

log [Per capita ASC density, volume, or utilization rate]

$$
\begin{aligned}
&= \beta_0 \\
&+ \beta_1\,(SES) \\
&+ \beta_2\,(y) \\
&+ \beta_3\,(SES \times y) \\
&+ \beta_4\,(Median\ age) \\
&+ \beta_5\,(Percentage\ of\ residents\ who\ are\ female) \\
&+ \beta_6\,(Percentage\ of\ residents\ who\ are\ Non-Hispanic\ White) \\
&+ \beta_7\,(Mean\ Charlson\ comorbidity\ score\ among\ all\ ambulatory\ surgery\ patients)
\end{aligned}
$$

y – year (continuous variable)
SES – Socioeconomic advantage score tertile (categorical variable with three levels)

© 2022 Chatterjee A et al. *JAMA Health Forum.*

References

1. Hall MJ, Schwartzman A, Zhang J, Liu X. Ambulatory Surgery Data From Hospitals and Ambulatory Surgery Centers: United States, 2010. Natl Health Stat Rep. 2017;(102):1-15.
2. Hollingsworth JM, Krein SL, Ye Z, Kim HM, Hollenbeck BK. Opening of Ambulatory Surgery Centers and Procedure Use in Elderly Patients: Data From Florida. *Arch Surg*. 2011;146(2):187-193. doi:10.1001/archsurg.2010.335
3. Strope SA, Sarma A, Ye Z, Wei JT, Hollenbeck BK. Disparities in the use of ambulatory surgical centers: a cross sectional study. *BMC Health Serv Res*. 2009;9(1):121. doi:10.1186/1472-6963-9-121
4. Janeway MG, Sanchez SE, Chen Q, et al. Association of Race, Health Insurance Status, and Household Income With Location and Outcomes of Ambulatory Surgery Among Adult Patients in 2 US States. *JAMA Surg*. 2020;155(12):1123-1131. doi:10.1001/jamasurg.2020.3318
5. Roux AVD, Merkin SS, Arnett D, et al. Neighborhood of Residence and Incidence of Coronary Heart Disease. *N Engl J Med*. 2001;345(2):99-106. doi:10.1056/NEJM200107123450205

© 2022 Chatterjee A et al. *JAMA Health Forum.*
